# Carbon Catabolite Repression Gene *AoCreA* Regulates Morphological Development and Ochratoxin A Biosynthesis Responding to Carbon Sources in *Aspergillus ochraceus*

**DOI:** 10.3390/toxins12110697

**Published:** 2020-11-03

**Authors:** Gang Wang, Yulong Wang, Bolei Yang, Chenxi Zhang, Haiyong Zhang, Fuguo Xing, Yang Liu

**Affiliations:** 1 Key Laboratory of Agro-Products Quality and Safety Control in Storage and Transport Process, Ministry of Agriculture and Rural Affairs, Institute of Food Science and Technology, Chinese Academy of Agricultural Sciences, Beijing 100193, China; wanggang02@caas.cn (G.W.); wylbiotech@126.com (Y.W.); yangbolei@caas.cn (B.Y.); zhangchenxi@caas.cn (C.Z.); zhanghaiyongwork@163.com (H.Z.); 2 School of Food Science and Engineering, Foshan University, Foshan 528231, China

**Keywords:** *Aspergillus ochraceus*, mycotoxin, carbon sources, carbon catabolite repression, CreA, Ochratoxin A

## Abstract

Carbon is one of the most important nutrients for the development and secondary metabolism in fungi. CreA is the major transcriptional factor mediating carbon catabolite repression, which is employed in the utilization of carbon sources. *Aspergillus ochraceus* contaminates various food and feed containing different carbon sources by producing ochratoxin A (OTA). However, little is known about the function of *AoCreA* in regulating the morphology and OTA production of *A. ochraceus*. To give an insight into the mechanism of the carbon sources regulating development of *A. ochraceus* and OTA production, we have identified *AoCreA* in *A. ochraceus*. The homologous recombination strategy was used to generate the *AoCreA* deletion mutant (Δ*AoCreA*). We have investigated the morphology and OTA production of the wild type (WT) and Δ*AoCreA* of *A. ochraceus* with media containing different carbon sources (glucose, fructose, maltose, D-xylose, D-mannose, acetate, D-galactose, D-mannitol and lactose). Δ*AoCreA* showed a significant growth and conidiation defect on all media as compared with WT. Glucose and maltose were the most inducing media for OTA production by *A. ochraceus*, followed by sucrose and the nutrient-rich Yeast Extract Sucrose (YES) and Potato Dextrose Agar (PDA). The deletion of *AoCreA* led to a drastic reduction of OTA production on all kinds of media except PDA, which was supported by the expression profile of OTA biosynthetic genes. Furthermore, infection studies of Δ*AoCreA* on oats and pears showed the involvement of *AoCreA* in the pathogenicity of *A. ochraceus*. Thus, these results suggest that *AoCreA* regulates morphological development and OTA biosynthesis in response to carbon sources in *A. ochraceus*.

## 1. Introduction

Ochratoxin A (OTA) is a secondary metabolite produced by different filamentous fungi belonging to the *Aspergillus* and *Penicillium* genera, and *A. ochraceus* is one of the main producer species [1]. OTA contamination of plant products such as barley, wheat, bread, beer, wine and coffee beans has become a serious health hazard worldwide [2]. It was also proved to have nephrotoxic, immunosuppressive, teratogenic and carcinogenic properties in animals and humans [2,3]. Several metabolites in the ochratoxin family were identified including OTA, OTB, OTC, OTα and OTβ [4,5,6], of which OTA has been shown to be the most toxic, and for this reason, it is widely studied. In 1993, OTA was listed as a possible carcinogenic substance (group 2B) by the International Agency for Research on Cancer [7]. Based on a comprehensive understanding of the dangers of OTA, researchers have paid more attention to the biosynthetic and regulatory mechanism of the toxin in the producer species [8,9,10,11,12,13].

Carbon is one of the most important nutrients for the growth and development of fungi; it also affects the production of secondary metabolites [14,15]. This leads to differences in mycotoxin production among different carbon source components. The food substrate containing the preferred carbon source for the fungal species will most likely be contaminated by mycotoxin. In the utilization of carbon sources by fungi, a mechanism known as carbon catabolite repression (CCR) is used to regulate the utilization of carbon sources. It results in the repression of enzymes required for the utilization of less favored carbon sources when more favored carbon sources are present in the medium [16,17,18]. In some *Aspergillus* species, D-glucose, sucrose, D-xylose and acetate are the most favored carbon sources and have the maximum inhibition ability against other carbon sources, while D-mannose, D-fructose, D-galactose and maltose are medium favored carbon sources, and lactose and L-arabinose are not favored carbon sources [19].

The CCR mechanism is regulated by a group of genes conserved in filamentous fungi including *CreA*, *CreB* and *CreC* [20]. In fungi, *CreA* is a transcription repressor containing two zinc fingers; it recognizes and binds to the 5′-SYGGRG-3′ (S = C/G, Y = C/T, R = A/G) on the promoter region of related genes [21]. The regulatory function of *CreA* on morphology and secondary metabolism was identified in many fungal species by constructing the gene deletion mutants. In *A. flavus*, the absence of *CreA* led to the inhibition of aflatoxin production on the complete medium, and Δ*CreA* could not effectively produce aerial hyphae on a minimal medium supplemented with different carbon sources [21]. In *A. niger*, *CreA* deletion mutants showed decreased growth rate and reduced spore production [19]. In *A. oryzae*, the production of α-amylase was improved after the deletion of genes *CreA* and *CreB* [22]. In *A. nidulans*, *CreA* has been shown to be functionally involved in the regulation of proline, ethanol, xylan and arabinan utilization [19]. *CreA* homologue genes were identified in other fungi species, such as *Mig1p* in *Saccharomyces cerevisiae* and *CaMIG1* in *Candida albicans* [23,24]. This protein has been implicated in the glucose repression of several genes in *S. cerevisiae.* However, disruption of the *CaMIG1* in *C. albicans* had no effect on filamentation or infectivity. It was also reported that in *Fusarium proliferatum*, the FUM gene cluster regulated the production of fumonisins in response to different carbon sources [25], suggesting that a possible role of *CreA* is also in this fungal species. Therefore, *CreA* plays important roles in fungi in response to carbon sources.

Although the role of the *CreA* gene regulating carbon catabolism in fungi has been extensively studied, little is known about the function of the *CreA* gene in *A. ochraceus*. In this study, we elucidate the functional role of *AoCreA* gene in the development, OTA production and pathogenicity in *A. ochraceus*.

## 2. Results

### 2.1. Identification of AoCreA in A. ochraceus

**Figure 1:**
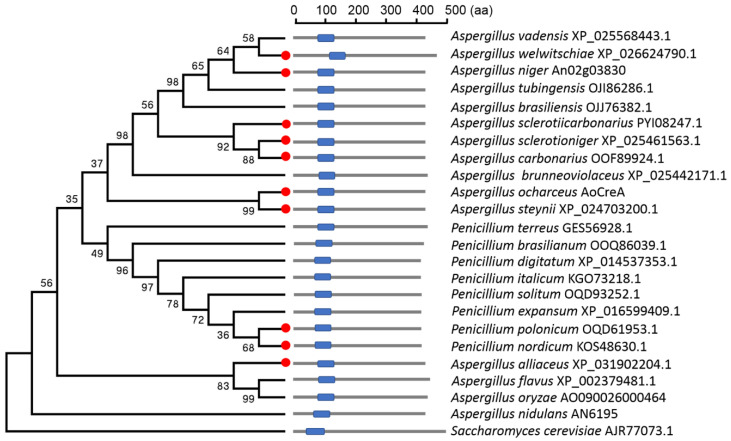
Phylogenetic relationship and structural domain analysis of the AoCreA homologs from different fungal species. The ochratoxin A (OTA)-producing fungi were marked in red. The blue boxes represent the C_2_H_2_ functional domain.

The major transcription repressor gene *AoCreA* of *A. ochraceus*, encoding of 427 amino acids, was identified based on protein BLAST with the CreA homologous proteins of *S. cerevisiae* (AJR77073.1) and *A. nidulans* (AN6195). A total of 24 homologous proteins from fungal species (15 *Aspergillus species*, 8 *Penicillium* species and 1 *Saccharomyces* species) were used to construct the phylogenetic tree. As shown in Figure 1, CreA from *A. steynii* (XP_024703200.1) was most related to AoCreA from *A. ochraceus*, with the identity of 97.4%. The length of the CreA protein was similar in different fungal species, which ranged from 400 to 500 amino acids, and the location of the C_2_H_2_ function domains were also similar. Furthermore, CreA proteins from OTA-producing fungi were not clustered in one branch, and CreA proteins from the same genus spread across different branches.

### 2.2. Identification of AoCreA Gene Disruption in A. ochraceus

**Figure 2:**
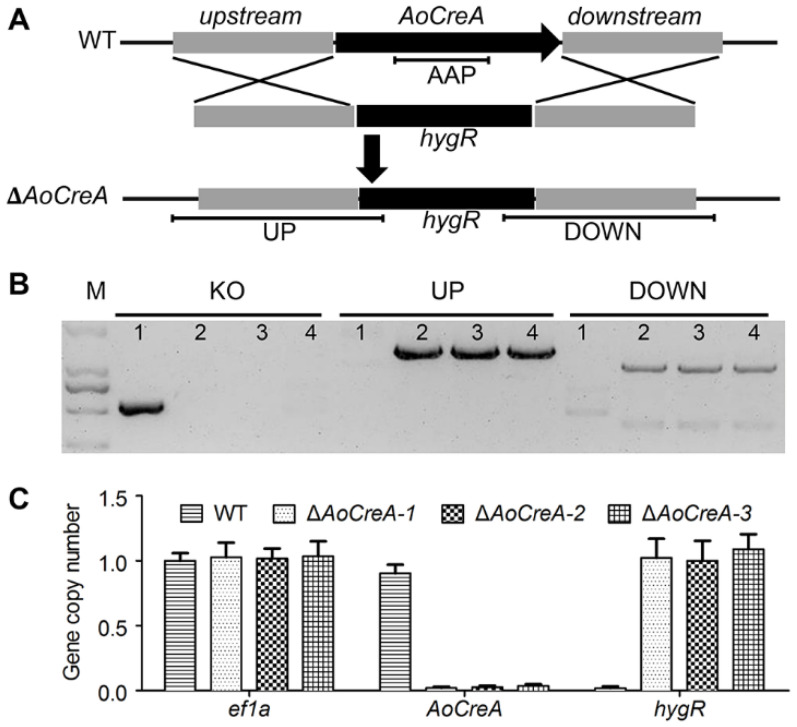
Obtainment of *A.* *ochraceus* Δ*AoCreA* strains. (**A**) Strategy for deletion of *AoCreA* in *A. ochraceus*. (**B**) PCR identification of *A. ochraceus* WT and Δ*AoCreA* mutants. Each primer pairs corresponds to one WT (1) and three Δ*AoCreA* mutants (2, 3 and 4). (**C**) Real-time genomic PCR analyses determine the gene copy numbers.

The *AoCreA* gene in *A. ochraceus* was replaced by *hygR* through the homologous recombination strategy (Figure 2A). A total of 64 mutant strains were first screened by a Potato Dextrose Agar (PDA) medium supplemented with 70 μg/mL of hygromycin, in which Δ*AoCreA* mutants could grow and WT could not. *A. ochraceus* WT and Δ*AoCreA* mutants were verified by diagnostic PCR. Finally, five transformants were verified to be positive, and the gene replacement efficiency by homologous recombination obtained was 7.8%. As shown in Figure 2B, the *AoCreA* amplification product (AAP) was detected in WT, which could not be detected in the three Δ*AoCreA* mutants. To identify whether the replacement of *hygR* was inserted in the right loci, the fragments UP and DOWN (Figure 2A) were both amplified. The results showed that UP and DOWN could be detected in Δ*AoCreA* mutants while not being detected in WT. In order to determinate the copy numbers of *hygR* construct that were integrated in the genome, real-time genomic PCR analyses were carried out. As a result (Figure 2C), both deletion mutants (Δ*AoCreA-1,* Δ*AoCreA-2* and Δ*AoCreA-3*) and WT have one copy of *ef1a* (reference gene). The WT has one copy of gene *AoCreA*, while the mutants do not have *AoCreA*. It was also found the three mutants have one *hygR* copy integrated within their genomes. Thus, no *hygR* construct ectopic insertions affect the Δ*AoCreA* phenotypes. The three mutants (Δ*AoCreA-1,* Δ*AoCreA-2* and Δ*AoCreA-3*) were regarded as three biological replicates of Δ*AoCreA* in the subsequent experiments.

### 2.3. Growth and Conidiospore Production Were Affected by Carbon Sources and Modulated by AoCreA

**Figure 3:**
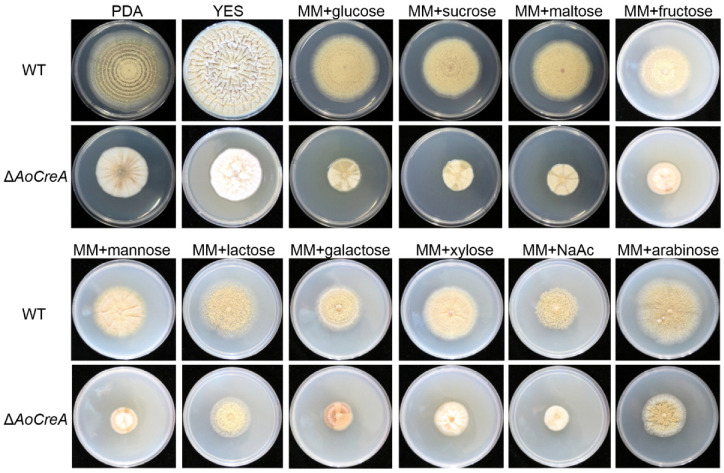
Colony view of *A. ochraceus* WT and Δ*AoCreA* grown on media containing different carbon sources. Both WT and Δ*AoCreA* had three biological replicates, and each strain was cultured on four plates as technical replicates.

**Figure 4:**
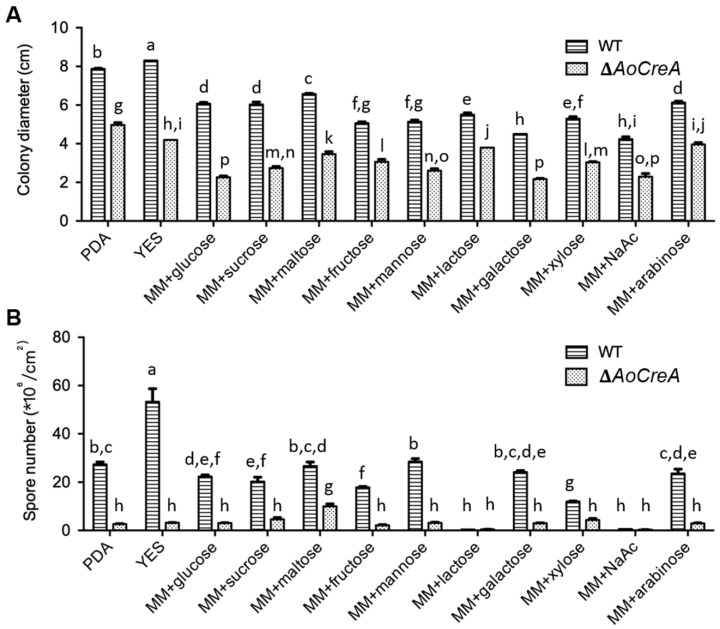
Effect of *AoCreA* deletion on the growth rate and conidiation of *A. ochraceus*. (**A**) Colony diameter of *A. ochraceus* WT and Δ*AoCreA* on different carbon sources. (**B**) Conidiospore production in *A. ochraceus* WT and Δ*AoCreA*. The error bars represent mean +/− SEM. Different letters indicate a significant difference between the corresponding values (*p* < 0.05) with three biological replicates.

A series of morphological changes were observed between *A. ochraceus* WT and three Δ*AoCreA* mutants when grown in different carbon sources (Figure 3). In PDA and Yeast Extract Sucrose (YES) media, conidiospores were largely produced in WT but not in Δ*AoCreA*. In the YES medium, as expected, wrinkles appeared on the surface of WT colony, while they disappeared in Δ*AoCreA*. When glucose, sucrose and maltose were used as carbon sources, the *A. ochraceus* WT and Δ*AoCreA* mutants showed almost the same morphology. When arabinose, NaAc and lactose were used as carbon sources, the colonies were transparent as little hypha was grown on the medium. The growth rate of *A. ochraceus* WT and Δ*AoCreA* grown in a single carbon source medium was slower than that grown on the YES and PDA media (Figure 3 and Figure 4A). The deletion of the *AoCreA* gene has a significant impact on the growth rate in all kinds of culture media (Figure 4A). In the media containing a single carbon source, glucose, sucrose, maltose and arabinose promote the growth of *A. ochraceus,* but the hypha were sparse in arabinose (Figure 3). The deletion of the *AoCreA* gene in *A. ochraceus* had a significant impact on the production of conidiospores. The deletion of *AoCreA* decreased the number of conidiospores on all kinds of media (Figure 4B). With lactose and NaAc as carbon sources, hardly any conidiospores were observed in WT and Δ*AoCreA*.

### 2.4. AoCreA Was Involved in OTA Biosynthesis

**Figure 5:**
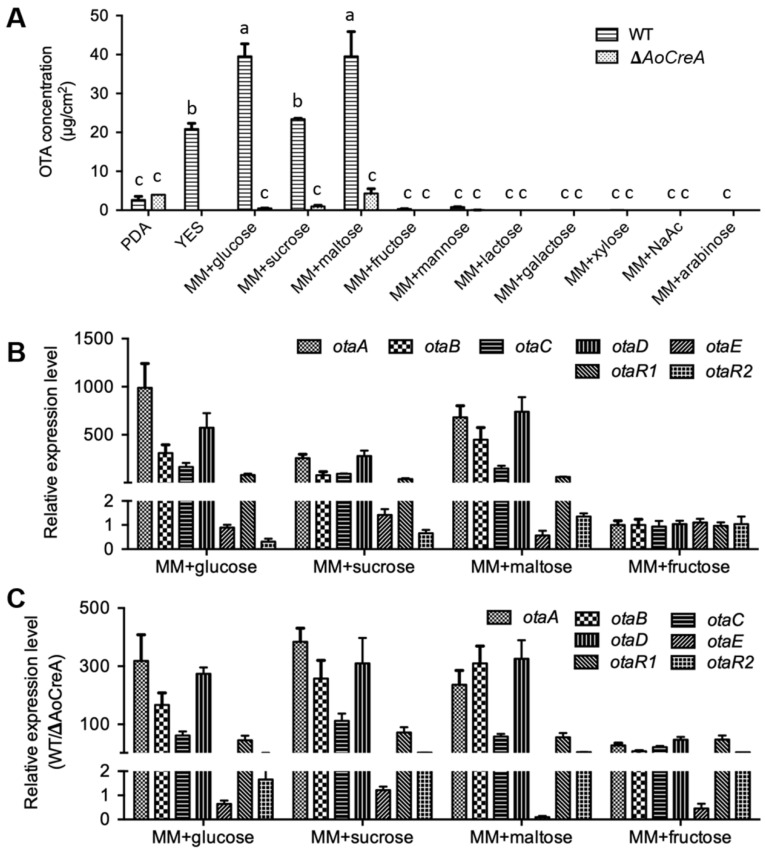
OTA biosynthesis in WT and Δ*AoCreA* strains of *A. ochraceus*. (**A**) OTA production of WT and Δ*AoCreA* on media containing different carbon sources. Different letters indicate a significant difference between the corresponding values (*p* < 0.05) with three biological replicates. (**B**) Relative expression level of OTA biosynthetic genes of WT on media containing glucose, sucrose, maltose and fructose analyzed by RTqPCR. (**C**) The expression ratio (WT/Δ*AoCreA*) of OTA biosynthetic genes on media containing glucose, sucrose, maltose and fructose. The ratios for different conditions demonstrated an extensive range, so the breakpoint was inserted into the *Y* axis. The error bars represent mean +/− SEM.

To investigate whether *AoCreA* is linked to the biosynthesis of OTA in *A. ochraceus*, OTA production was analyzed by High-Performance Liquid Chromatography (HPLC). The samples were collected after 12 days of cultivation at 28 °C under dark conditions and minimal media (MM) containing different carbon sources; PDA and YES were used as the cultural media. The results showed that glucose, sucrose and maltose contribute more to the biosynthesis of OTA. The concentration of OTA reached around 40 μg/cm^2^ for the MM containing glucose and maltose, around 20 μg/cm^2^ for the YES medium and MM containing sucrose and around 4 μg/cm^2^ for the PDA medium. Conversely, in other carbon sources, little or no OTA was detected (Figure 5A). These results demonstrated that glucose and maltose were more suitable for *A. ochraceus* to produce OTA, followed by sucrose and the nutrient-rich YES and PDA. OTA production was suppressed by D-xylose, D-mannose, acetate, D-galactose, D-mannitol, lactose and L-arabinose. In Δ*AoCreA* mutants, the production of OTA was drastically reduced compared with WT on all the media except PDA.

Seven genes (*otaA*, *otaB*, *otaC*, *otaD*, *otaE*, *otaR1* and *otaR2*) consisting of a gene cluster were involved in the biosynthesis of OTA in *A. ochraceus,* as reported previously [9]. The relative expression level of these genes in *A. ochraceus* grown on media containing OTA-induced carbon sources (glucose, sucrose and maltose) and an OTA-suppressed carbon source (fructose) was examined (Figure 5B). The expression level of five genes (*otaA*, *otaB*, *otaC*, *otaD* and *otaR1*) increased drastically when *A. ochraceus* was cultured on the media containing OTA-induced carbon sources compared with being cultured on the OTA-suppressed carbon source. The expression level changes were not obvious for the genes *otaE* and *otaR2*. The gene expression level ratio of WT to Δ*AoCreA* was also calculated as shown in Figure 5C. The expression of five genes (*otaA*, *otaB*, *otaC*, *otaD* and *otaR1*) were drastically regulated by *AoCreA* when *A. ochraceus* was cultured on all the media tested. In more detail, the deletion of the *AoCreA* gene led to a 300-fold reduction of expression of *otaA* when *A. ochraceus* was cultured on a medium containing glucose. In particular, the expression ratio of the seven genes showed a similar profile when WT and Δ*AoCreA* were cultured on the OTA-suppressed medium containing fructose.

### 2.5. AoCreA in A. ochraceus Is Required for Fungal Infection

**Figure 6:**
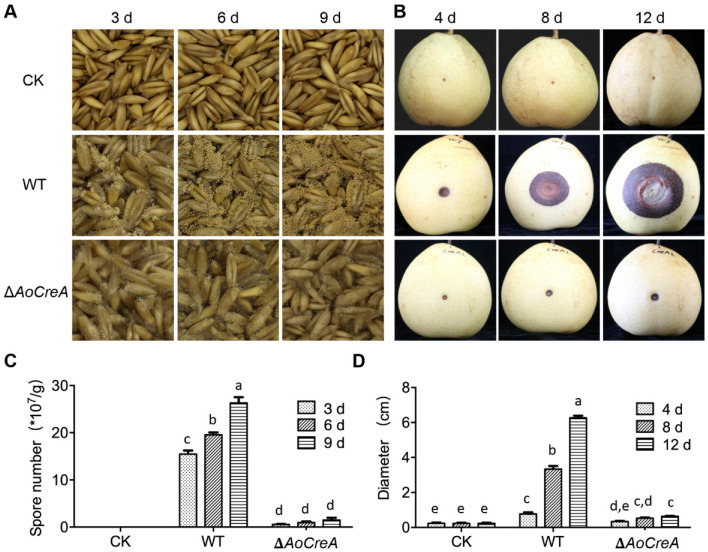
Pathogenicity assay for WT and Δ*AoCreA* of *A. ochraceus* on oats and pears. (**A**) Infection of *A. ochraceus* WT and Δ*AoCreA* on oats. (**B**) Infection of *A. ochraceus* WT and Δ*AoCreA* on pears. (**C**) Conidiospore production of *A. ochraceus* grown on oats. (**D**) Diameter of lesion on pears. Different letters indicate a significant difference between the corresponding values (*p* < 0.05) with three biological replicates. The error bars represent mean +/− SEM.

The role of the *AoCreA* gene of *A. ochraceus* in the pathogenicity was studied through infection experiments on oats and pears. The phenotype of oats was measured at 3, 6 and 9 days after inoculation (Figure 6A). In detail, it was observed that lots of conidiospores on the surface of oats were inoculated with WT with respect to those inoculated with Δ*AoCreA*. Additionally, the number of conidiospores on oats infected by WT increased over time. When oats were infected by Δ*AoCreA*, the conidiospore number was maintained at a low level (Figure 6C). In pear infections, the phenotype of pears was measured at 4, 8 and 12 days after inoculation (Figure 6B,D). The lesion, as a consequence of WT infection, increased over time, while that of Δ*AoCreA* was limited and was without expansion over time.

## 3. Discussion

Studies about the ochratoxin A (OTA) contamination in food safety and its biosynthetic process had been well studied in OTA producing fungi, but little was known about *AoCreA* regulated carbon source utilization in the biosynthesis of OTA in *A. ochraceus*. Carbon source is a kind of basic energy source in the growth and development of fungi, and it also plays an important role in the biosynthesis of secondary metabolites [15]. The contamination of mycotoxin in food and feed depends mostly on the types of nutrients in food substrates. Thus, most fungi have their preference during infection. This is also reflected in different stages of crop development, as the preferred carbon source may be massively synthesized during one stage. For example, maize is vulnerable to aflatoxin contamination during late development [26]. From this point of view, mycotoxin can be controlled by modifying the components of the carbon source in crops through cultivating new types of crop varieties. Many studies showed that many kinds of physical phenotypes such as growth rate, conidia production and secondary metabolite production were influenced by the utilization of a carbon source. CreA was an important regulator in this process. Furthermore, few studies have explored how CreA is regulated at the level of transcription and post-translation. Phosphorylation was demonstrated to be a post-translational pathway for regulation of CreA in *S. cerevisiae* and *Trichoderma reesei* through the involvement of the protein kinase Snf1p in the process [27,28]. CreA was also found to be regulated by ubiquitination. In fact, when cell signal was sensed by the CreB-CreC de-ubiquitination complex (DUB), CreA was de-ubiquitinated and activated, followed by CreA activating as a transcription factor in carbon catabolite repression (CCR) [29].

In our study, phylogenetic analysis indicated the function of CreA was conserved during evolution. The results illustrated that the utilization of different carbon sources was not the same in *A. ochraceus*; the *AoCreA* gene played an important role in the growth and conidiation in *A. ochraceus*. The *AoCreA* gene also played an important role in the biosynthesis of OTA in *A. ochraceus*, and the regulatory role of *AoCreA* responded to carbon sources. Furthermore, the deletion of *AoCreA* led to the weakened pathogenic ability of *A. ochraceus*. *A. ochraceus* has the maximum capacity for growth, conidiation and OTA production on the MM containing glucose, sucrose or maltose, which are the most abundant in the natural environment. This may be the result of adaptation of the environment during evolution. *A. ochraceus* infected pear and oat with severe symptoms under natural conditions, while the pathogenicity greatly decreased in the *AoCreA* deletion mutant. Glucose was abundant in the pear, and the starch in the oat would be digested to glucose by amylase in *A. ochraceus* [30]. These results illustrate that *AoCreA* plays an important role in the development of *A. ochraceus,* and many metabolic pathways were regulated by the gene. The genome of *A. ochraceus* was published in 2018 and consensus of the OTA biosynthetic pathway was found by a comparative genomic analysis of six OTA-producing fungi [9]. Additionally, five genes representing a putative OTA-gene cluster (*otaA*, *otaB*, *otaC*, *otaD* and *otaR1*) showed a consistent expression pattern. For the genes *otaE* and *otaR2* located close to the OTA-putative gene cluster, the expression pattern under different carbon sources was different. This result could be an evidence for the unnecessary role of the two genes in OTA biosynthesis, confirming the results obtained for *A. carbonarius*, another main OTA-producing fungus and other OTA-producing fungi [11,12].

## 4. Conclusions

In conclusion, the *AoCreA* gene regulates morphological development and OTA biosynthesis responding to carbon sources in *A. ochraceus*. As far as we know, this is the first report about *AoCreA* in *A. ochraceus*. As an important global regulatory factor, there are still more functions to be explored, and how *AoCreA* functions in the CCR process is still unknown. This study provides some evidence about *AoCreA* in the development of *A. ochraceus*, which may provide some theoretical basis in the prevention of *A. ochraceus*.

## 5. Materials and Methods

### 5.1. Strains and Culture Conditions

The WT *A. ochraceus* used in this study was stored in Institute of Food Science and Technology, Chinese Academy of Agricultural Sciences [9]. The fungal strains used in this study were cultured at 28 °C in dark conditions. Potato Dextrose Agar (PDA, Becton, Dickinson and Company, Franklin Lakes, NJ, USA) and Yeast Extract Sucrose (YES, 5% yeast extract, 40% sucrose and 10% agar) were routinely used to culture *A. ochraceus* strains. Minimal media (MM, 6.0 g NaNO_3_, 0.52 g KCl, 0.52 g MgSO_4_·7H_2_O, 1.52 g KH_2_PO_4_, 1 mL trace elements, 10 g glucose, 12.5 g agar, pH 6.5, in 1 L distilled water) [31], supported with different carbon sources at the concentration of 2%, were used to detect the response of *A. ochraceus*. The test for the evaluation of different carbon sources included the adding to the MM of glucose, fructose, maltose, D-xylose, D-mannose, acetate, D-galactose, D-mannitol, lactose and L-arabinose. Each experiment was performed three times as biological replicates.

### 5.2. AoCreA Identification and Phylogenetic Analysis

CreA amino acid sequences from *S. cerevisiae* (AJR77073.1) and *A. nidulans* (AN6195) were used as queries, and the Basic Local Alignment Search Tool (BLAST, NCBI, Bethesda, MD, USA) algorithm was used to search AoCreA from the genome of *A. ochraceus*. Other CreA sequences were downloaded from the National Center for Biotechnology Information resources (NCBI). The amino acid sequences of CreA were aligned by ClustalW, and a maximum likelihood phylogeny was constructed by MEGA 5.1 software [32] using 1000 bootstrap replicates.

### 5.3. Construction of Gene Deletion Mutants

The construction of the *A. ochraceus AoCreA* deletion mutant was performed using overlap PCR procedures, which were previously described [9]. A total of 776 bp from the whole *AoCreA* gene (1284 bp) in the *A. ochraceus* genome was designed to be replaced by *hygR* (hygromycin B phosphotransferase). Primers used in this study are listed in Table S1. The fusion PCR products with the *hygR* marker were then transformed into the protoplasts of *A. ochraceus*. Positive transformants were verified by diagnostic PCR [33]. And real-time genomic PCR amplification was used to analyze the copy number of *hygR* constructs, as previously described [10,34]. Briefly, genomic DNA from each of the strains was extracted and diluted to 10 ng/μL. qPCR reactions were performed using SYBR Green supermix (Takara, Kyoto, Japan) on a Applied Biosystems 7500 Real Time PCR system (Life Technologies, Waltham, MA, USA). The number of gene copies was calculated depending on the Ct (Cycle threshold) value, with an elongation factor 1α (ef1a) as the single copy reference gene [34]. The primers used are listed in Table S1.

### 5.4. Phenotypic Studies

In order to study the effects of *AoCreA* deletion on vegetative growth and asexual sporulation, WT and deletion mutants were inoculated on PDA, YES and MM supplies for different carbon sources (glucose, fructose, maltose, D-xylose, D-mannose, acetate, D-galactose, D-mannitol, lactose and L-arabinose). Plates were inoculated with 1 μL of conidiospore suspension (106 cfu/mL) and cultured at 28 °C in darkness for 12 days. The growth rate was measured by checking the diameter of each colony. For the sporulation study, every medium was washed with 10 mL of 0.1% Tween-80 solution, and the number of conidiospores was counted under a microscope with a hemocytometer.

### 5.5. DNA and RNA Extraction

To extract genomic DNA, the mycelium of *A. ochraceus* was harvested from a shaking culture in the PDB medium, 28 °C, 180 rpm/min for 3 days. The DNA was extracted using a DNeasy kit (Qiagen, Hilden, Germany), according to the manufacturer’s protocol. For RNA extraction, the mycelium was cultured on a solid culture medium for 12 days under dark conditions. RNA was extracted using the EASYspin Plus Plant RNA Kit (Aidlab, Beijing, China) following the manufacturer’s protocol. A total of 500 ng RNA were used for the synthesis of cDNA. The removal of gDNA and synthesis of first strand cDNA were performed using the FastQuant RT Kit (TIANGEN, Beijing, China).

### 5.6. OTA Extraction and Production Analysis

To detect the production of OTA, each medium of the *A. ochraceus* strain was cultured in 28 °C under dark conditions for 12 days. A quarter of the medium was carved and cut up to small pieces. The medium pieces were steeped with 20 mL of methanol for 1 h. Ultrasonic vibration and vortexing were used to make sure all the toxins had been dissolved into methanol. After 30 min of ultrasonic vibration and 3 min of vortexing, 1 mL of the supernatant solution was filtered through a 0.22 μm filter into a vial. For OTA detection, HPLC analysis was performed on an Agilent HPLC system (Agilent Technologies, Santa Clara, CA, USA) with a mobile phase of acetonitrile/water/acetic acid (99:99:2, *v*/*v*); each sample was running for 20 min, as previously described [9]. For each plate, the total amount of OTA divided by the colony area (μg/cm^2^) was used to express OTA concentration.

### 5.7. Gene Expression Analysis

RTqPCR amplification was performed using the SYBR Green supermix on the Applied Biosystems 7500 Real Time PCR system. The obtained results were normalized against the expression of the *GADPH* gene of *A. ochraceus*. The relative expression level was calculated using the 2^−ΔΔct^ method [35]. Primers used for gene expression are listed in Table S1.

### 5.8. Pathogenicity Assay

As *A. ochraceus* usually occurs in cereals and fruits, oats and pears were used as infection hosts to study the pathogenicity of *A. ochraceus* strains in this experiment. In the oat infection experiment, 20 g of oats were weighed and the water activity was adjusted to 0.95. Each bottle of oats was inoculated with 1 mL of 106 cfu/mL conidiospores and incubated at 28 °C. At 3, 6 and 9 days after inoculation, the conidiospores around the oats were washed with 0.1% Tween-80 solution and counted by a hemocytometer. In the pear infection experiment, the surface of each pear was disinfected with 75% alcohol. After the alcohol was volatilized, a hole of 3 mm in depth was punctured in the center of the pear with a sterilized toothpick. A measure of 1 μL of 10^6^ cfu/mL conidiospores were injected and incubated at 28 °C.

### 5.9. Statistical Analysis

All the data were analyzed with IBM SPSS statistics version 20 and presented with the means and +/− SEM of three biological replicates samples. Different letters were used to indicate statistically significant differences analyzed by ordinary one-way analysis of variance (ANOVA) Fisher’s LSD test (*p* < 0.05).

## Supplementary Materials

The following are available online at https://www.mdpi.com/2072-6651/12/11/697/s1, Table S1: Primers used in this study.
