# Supplementary material for: Carbon Catabolite Repression Gene AoCreA Regulates Morphological Development and Ochratoxin A Biosynthesis Responding to Carbon Sources in Aspergillus ochraceus
Source: Toxins (Basel). 2020 Nov 3;12(11):697. doi: 10.3390/toxins12110697 (PMC7693787; doi:10.3390/toxins12110697)
Supplement: Supplementary file 1 [file toxins-12-00697-s001.pdf]

Supplementary Materials: Carbon Catabolite Repression Gene *AoCreA* Regulates Morphological Development and Ochratoxin A Biosynthesis Responding to Carbon Sources in *Aspergillus ochraceus*

Gang Wang , Yulong Wang, Bolei Yang, Chenxi Zhang, Haiyong Zhang, Fuguo Xing and Yang Liu

Table S1. Primers used in this study.

Primers	Oligonucleotide sequence (5′-3′)	Uses
hygR-F	GGAGGTCAACACATCAATGCCTATT	*hygR* amplification
hygR-R	CTACTCTATTCCTTTGCCCT	
creA-up-F	CGGTCAGTCAGGACTCAGTAG	*AoCreA* deletion cassette
creA-up-R	ACCAAAATAGGCATTGATGTGTTGACCTCCACACTTGTAGGGTCGAGGGA	
creA-down-F	CACTCGTCCGAGGGCAAAGGAATAGAGTAGGATTCGCCATCTGTCGCTCC	
creA-down-R	CTACCATGTTCGCGCATTCTTC	
creA-KO-F	CCCGACACATTCGAACCCAC	Identification of *AoCreA* deletion mutants
creA-KO-R	GCTGTGCGAGATTGCGTAGG	
creA-up-check-F	CGGTCAGTCAGGACTCAGTAG	
HygR-up-check-R	GGCTGATCTGACCAGTTGCC	
HygR-down-check-F	GGCTGTGTAGAAGTACTCGCC	
creA-down-check-R	CTACCATGTTCGCGCATTCTTC	
GADPH-RT-F	CGGCAAGAAGGTTCAGTT	Primers used for detect the expression of OTA biosynthetic genes
GADPH-RT-R	CTCGTTGGTGGTGAAGAC	
otaR2-RT-F	CGATGCTACTGGAGACAA	
otaR2-RT-R	TCGATGCGTCTATCAAGG	
otaE-RT-F	CTGGGCATGCTTTCAAGT	
otaE-RT-R	GCACTGTCAACTTCCTCAA	
otaA-RT-F	GGATCTTTATGACCGAATCAG	
otaA-RT-R	CCTTGACCTGAAGAATGCT	
otaB-RT-F	ATACCACCAGAGCTCCAAA	
otaB-RT-R	GAGATGTTCGGTCTGTTCA	
otaC-RT-F	CTTAATACGGTGGTCTACGA	
otaC-RT-R	GAATGATAGGTCCGTATTTCT	
otaR1-RT-F	GCTTTCAAATCGAATGATTCC	
otaR1-RT-R	GATCGGTTGGAAGTGTAGAA	

otaD-RT-F	TATTCCCTAGATACCATATCG G	
otaD-RT-R	GCTTCCTTCTGGTTGTTCA	
ef1a-q-F	GTCTGGTGATGCTGCCATCG	Primers used for detect the copy of gene numbers
ef1a-q-R	CCTTGATGACACCGACAGCG	
AoCreA-q-F	CCCGACACATTCGAACCCAC	
AoCreA-q-R	CATGGCATTGTCTTGTCCGG	
hygR-q-F	GTCACGTTGCAAGACCTGCC	
hygR-q-R	CGCGCATATGAAATCACGCC	
